# Facial trustworthiness dampens own-gender bias in emotion recognition

**DOI:** 10.1007/s00426-023-01864-2

**Published:** 2023-08-09

**Authors:** Arianna Bagnis, Valentina Colonnello, Paolo Maria Russo, Katia Mattarozzi

**Affiliations:** Department of Medical and Surgical Sciences, University of Bologna, Sant’Orsola Hospital, Pad. 21, Bologna, Italy

## Abstract

Previous research suggests that emotion recognition is influenced by social categories derived by invariant facial features such as gender and inferences of trustworthiness from facial appearance. The current study sought to replicate and extend these findings by examining the intersection of these social categories on recognition of emotional facial expressions. We used a dynamic emotion recognition task to assess accuracy and response times in the happiness and anger categorization displayed by female and male faces that differed in the degree of facial trustworthiness (i.e., trustworthy- vs. untrustworthy-looking faces). We found that facial trustworthiness was able to modulate the own-gender bias on emotion recognition, as responses to untrustworthy-looking faces revealed a bias towards ingroup members. Conversely, when faces look trustworthy, no differences on emotion recognition between female and male faces were found. In addition, positive inferences of trustworthiness lead to faster recognition of happiness in females and anger in males, showing that facial appearance was able to influence also the intersection between social categories and specific emotional expressions. Together, these results suggest that facial appearance, probably due to the activation of approach or avoidance motivational systems, is able to modulate the own-gender bias on emotion recognition.

## Introduction

Faces are one of the most salient stimuli in social communication, as they provide information useful for social inference and shape efficient interactions (Jack & Schyns, 2015). This information is mainly based on the perception of invariant (e.g., facial structure, eye shape) and variant (e.g., facial expression) features of a face (Haxby et al., 2000; Quinn & Macrae, 2011). The literature suggests that social categories derived from invariant facial features, such as gender, interact with emotional expressions derived from variant facial features to influence interpersonal and intergroup behaviors (Bagnis et al., 2019; Craig & Lipp, 2017; Freeman & Johnson, 2016; Herlitz & Lovén, 2013; Hewstone et al., 2002; Macrae & Bodenhausen, 2000; Mason et al., 2006; Wacker et al., 2017).

Several studies reported an own-gender bias, especially in women that seem to be more accurate at recognizing female (i.e., ingroup stimuli) faces than male (i.e., outgroup stimuli) faces (Herlitz & Lovén, 2013; Lovén et al., 2011; Man & Hills, 2016; Rehnman & Herlitz, 2007). It has also been shown that happy facial expressions were recognized more quickly when shown by females, while anger was recognized more quickly when shown by males, regardless the gender of observers (Hess et al., 2004; Kret et al., 2011). This interaction between gender and discrete emotional expressions can be explained by two mutual accounts (Craig & Lee, 2020). The visual-structural account (bottom-up) suggests that the interaction is facilitated by an overlap between men's and women's facial features (e.g., square jaw, thicker eyebrows, a round face with large eyes) and angry and happy expressions, respectively (Becker et al., 2007). The stereotype-based account (top-down) suggests instead that this may be due to cultural stereotypes associating men with aggressiveness and women with more positive evaluations (Harris & Ciaramitaro, 2016; Hugenberg & Sczesny, 2006).

In social interactions, another important process led by invariant features from faces (i.e., facial appearance) is the automatic inference of a person's social traits, such as trustworthiness (Todorov et al., 2015). When personal information is not accessible, these inferences guide behavior, such as approaching and remembering trustworthy-looking faces more than untrustworthy-looking faces (Mattarozzi et al., 2015, 2017; Oosterhof & Todorov, 2008). Previous research has established that emotionally neutral faces rated as trustworthy are perceived as expressing happiness, while neutral faces rated as untrustworthy are perceived as expressing anger (Oosterhof & Todorov, 2008, 2009) because of an overgeneralization of adaptive mechanisms underlying the processing of emotional faces (Montepare & Dobish, 2003; Oosterhof & Todorov, 2008, 2009; Said et al., 2009). Recent studies investigating the relationship between facial trustworthiness and emotion recognition highlighted that trustworthy-looking faces enhance a general emotion recognition compared to untrustworthy-looking faces (Colonnello et al., 2019a, 2019b; Colonnello et al., 2019a, 2019b). This effect resonates with the fact that positive and negative inferences differentially activate motivational systems and behavioral responses, i.e., appetitive/approach and defensive/avoidance, leading to an advantage or a disadvantage on emotion recognition (Lang & Bradley, 2010).

Building upon extant research, we can hypothesize that gender would interact with facial appearance-based inferences of trustworthiness in emotion recognition. To date, no studies have investigated the intersection of social categories from different invariant facial features (i.e., gender and facial trustworthiness) influencing emotion recognition. Accordingly, we aimed to examine whether gender would affect emotion recognition as a function of facial trustworthiness. Specifically, we tested this hypothesis by measuring the accuracy and reaction times during an emotion recognition task while female participants watched dynamic emotional expressions (i.e., happiness and anger) displayed by female and male faces varying in level of facial trustworthiness (i.e., trustworthy- vs. untrustworthy-looking faces).

Since trusting someone is a crucial aspect that drives how people behave toward each other, we might expect facial trustworthiness to modulate gender bias in emotion recognition. Following Colonnello et al., (2019a, 2019b), it can be hypothesized that (H1a) trustworthy-looking faces would lead to an overall improvement in the recognition of emotional expressions by activating the appetitive motivational system, dampening the own- gender bias. Conversely, untrustworthy-looking faces would reveal an own-gender bias (H1b). As ingroup members makes people more inclined to approach them (Paladino & Castelli, 2008), even if they are untrustworthy, they would favor emotion recognition compared to untrustworthy outgroup members (i.e., untrustworthy male faces observed by women).

**Fig. 1:**
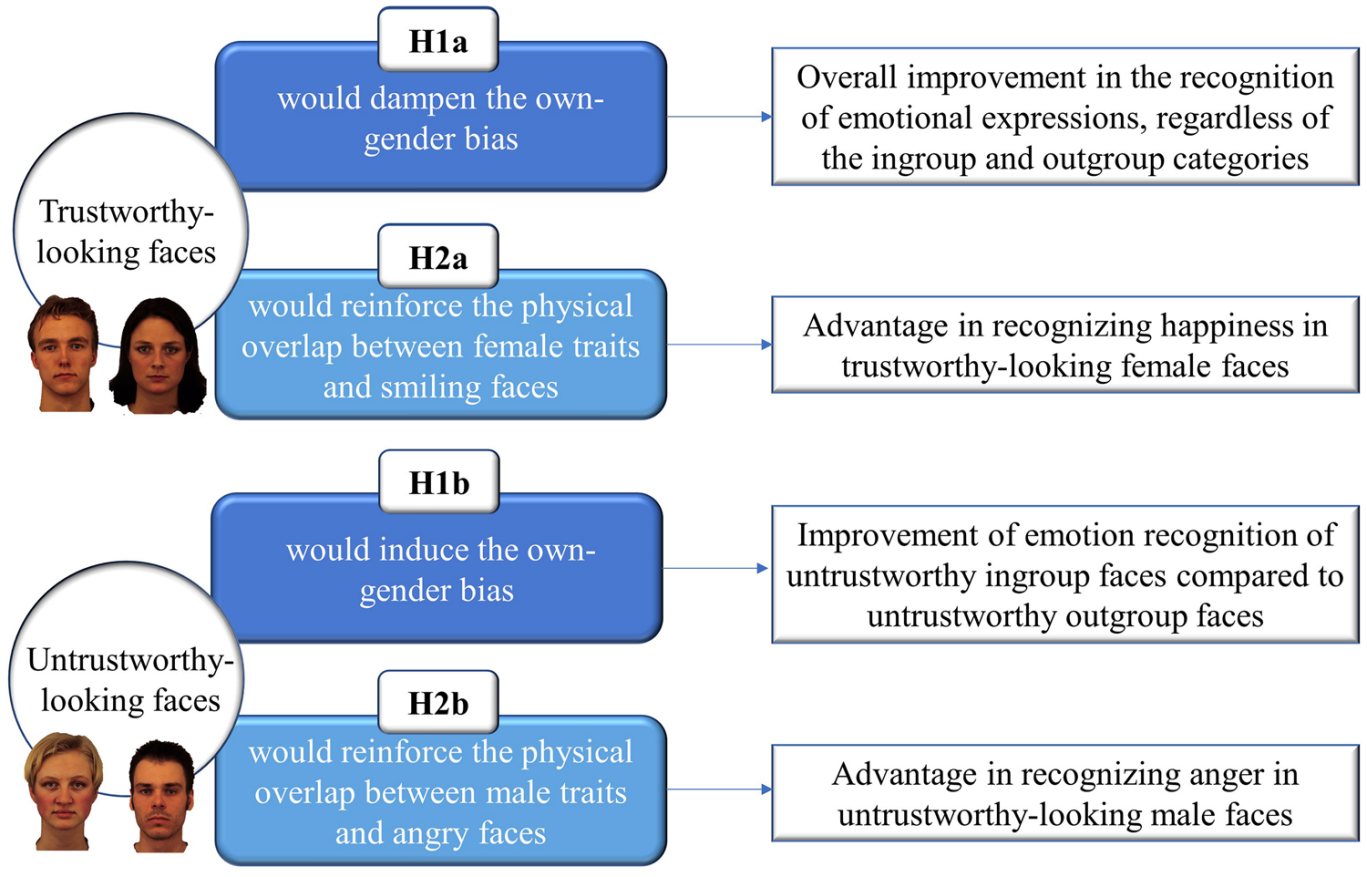
Visual summary of the hypotheses

In addition, in line with the visual structural and stereotype-based accounts (Craig & Lee, 2020), we might expect the results to differ according to emotional expressions, namely happiness and anger. Also, it is important to note that global face characteristics, such as a masculine/feminine appearance, influence inferences of trustworthiness, as feminine faces are usually judged as more trustworthy than masculine faces and vice versa (Hess et al., 2009; Oosterhof & Todorov, 2008; Todorov et al., 2015). These perceptual overlaps between the facial configuration of trustworthy-looking and feminine faces may contribute to the happy-female advantage (H2a), whereas the advantage in perceiving anger in males would be stronger for untrustworthy-looking targets than for trustworthy-looking targets (H2b), by reinforcing the well-known physical overlap between female/male facial traits and smiling/angry faces (Craig & Lee, 2020). See Fig. 1 for a visual summary of the hypotheses.

## Method

### Participants

A total of 109 White female individuals (mean ± SD age = 43.77 ± 9.46) with normal or corrected-to-normal visual acuity participated in this experiment. This sample exceed the minimum number (88 participants) needed to achieve a statistical power of 0.95 for alpha = 0.05, assuming a medium effect size and a correlation of 0.50 among repeated measures (Faul et al., 2007).

All participants were recruited from students or administrative staff from the School of Medicine. Written informed consent was obtained from all participants prior to the study, and a full debriefing was provided at the study’s conclusion. The experiment was conducted in accordance with the Declaration of Helsinki, and was approved by the Institutional Review Board (IRB) of the University of Bologna.

### Materials and procedure

A total of 48 video-clips (10 s each, 25 frames/s) were used as stimuli. Each video clip showed a neutral facial expression gradually and continuously changing into a basic full-intensity facial emotional expression (happiness, anger). To build them, 72 frontal color photographs of the faces of 12 Caucasian actors were used. 6 female and 6 male actors were selected from the Karolinska Directed Faces Database (Lundqvist et al., 1998,). The images were selected based on a standardized average (z score) of their trustworthiness ratings, as in Oosterhof and Todorov (2008). Specifically, from the database available at https://tlab.uchicago.edu/databases/, we selected the three male (z =  + 0.95 ± 0.11; faces: AM43, AM58, AM66) and three female (z =  + 1.09 ± 0.22; faces: AF06, AF19, AF01) faces rated as the most trustworthy-looking (*t*(4) = 0.958, *p* = 0.196), and the three male (z = 0.01 ± 0.02; faces: AM42, AM67, AM68) and three female (z = 0.11 ± 0.14; faces: AF12, AF21, AF33) faces rated as the most untrustworthy-looking (*t*(4) = 1.419, *p* = 0.114). The images used for the practice trials had neutral trustworthiness z scores (z =   −  0.04 ± 0.2, faces: AM44; AF04).

For each actor, we selected images representing a neutral emotional expression and two full-intensity emotional expressions (happiness, anger). Two additional images presenting the neutral and full emotional expressions of two actors were used to construct the videos for the practice trials.

Each image was manipulated to delete extraneous attributes (e.g., hair) and subjected to morphing by means of FantaMorph© software (Abrosoft, 2011http://www.fantamorph.com/index.html). First, for each actor, morph sequences with increasing emotional intensity were created based on two images: a neutral face as the first frame, and a full emotional face (happy, anger) as the final frame. Then, for each actor, two video-clips (neutral-happy, neutral-angry) were composed.

Participants were seated in front of the computer screen on which the video-clips were presented and responded using the computer keyboard. They received oral and written instructions and were given four practice trials before the experiment started. The task consisted of 48 trials and each of them was preceded by a central fixation cross. The video-clips presentation order was pseudorandomized controlling for gender, trustworthiness, and emotion. The total duration of the task was ~ 20 min.

Participants were instructed to view each video and press the keyboard spacebar as soon as they felt certain that the image contained more of the features of a specific emotion than of the initial neutral facial expression. Immediately after stopping the video, the stopped frame remained visible on the center screen and the participant identified the displayed emotion by completing a forced-choice task recognition between two possible emotion labels (happiness, anger). Recognition accuracy and response times were recorded.

For stimulus presentation and response data collection, we used E-Prime software (Psychology Software Tools, Pittsburgh, 2016).

### Statistical analysis

The accuracy (i.e., the percentage of correct responses) and the response time (i.e., the time in ms required to correctly recognize the emotions) data were analyzed using separated repeated-measures ANOVAs, with Face Gender (ingroup/female, outgroup/male), Facial Trustworthiness (trustworthy, untrustworthy) and Emotion (happiness, anger) as within-subject factors, followed by post-hoc Bonferroni-corrected comparisons. In line with prior research showing that recognition of emotions (e.g., Mill et al., 2009) decreases in older people, we controlled for age.

Effect sizes were calculated using partial eta squared. The alpha level for all analyses was set to *p* < 0.05. All the analyses were run using SPSS version 25.0 (IBM Corp, 2020, Chicago, IL).

## Results

### Accuracy

**Fig. 2:**
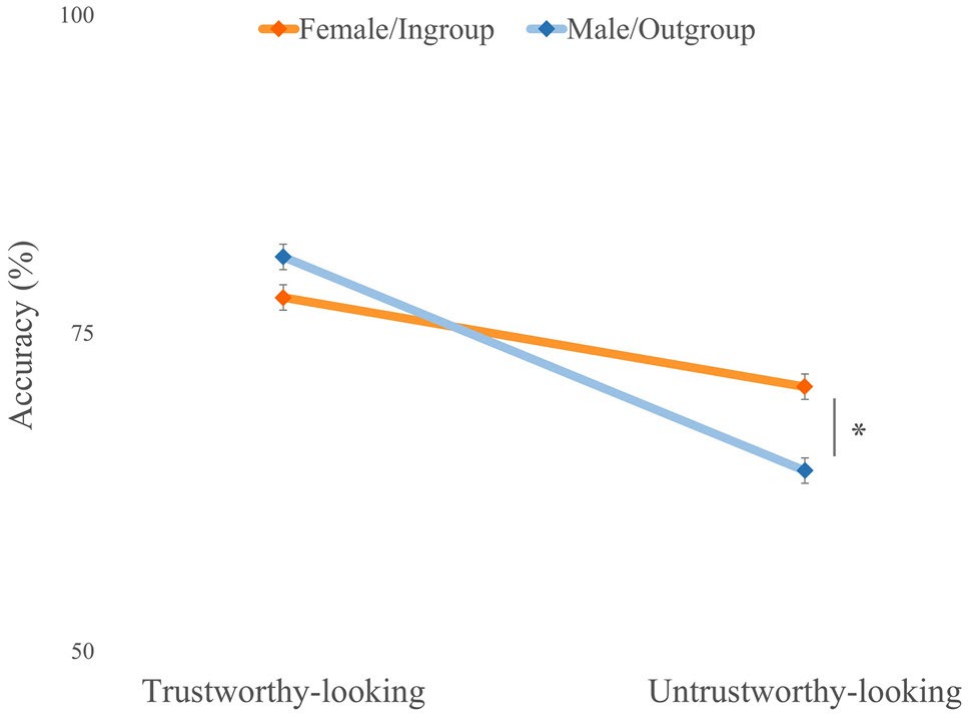
Accuracy (%) for the emotion recognition on female and male targets in trustworthy-looking and trustworthy-looking faces. Error bars represent standard error of the mean. Note. ∗p < 0.001

Results showed a main effect of Facial Trustworthiness,* F*(1, 108) = 76.13, *p* < 0.001, *η*^*2*^_*p*_ = 0.41, with trustworthy-looking faces (*M* = 80%, *SD* = 20%) being recognized significantly more accurately than untrustworthy-looking faces (*M* = 67%, *SD* = 21%). A main effect of Emotion was found, showing that emotion recognition was more accurate for happiness (*M* = 87%, *SD* = 21%) compared to anger (*M* = 59%, *SD* = 26%), *F*(1, 108) = 128.65, *p* < 0.001, *η*^*2*^_*p*_ = 0.54. Interestingly, there was an interaction between Face Gender and Facial Trustworthiness, *F*(1, 108) = 11.99, *p* < 0.001, *η*^*2*^_*p*_ = 0.10. Since we had clear predictions that intergroup bias in emotion recognition would be influenced by facial trustworthiness, we made planned comparisons to compare the recognition performance for emotional expressions from trustworthy-looking faces displayed by female (i.e., ingroup) vs. male faces (i.e., outgroup) and from untrustworthy-looking faces displayed by female vs. male faces (i.e., outgroup). Post-hoc Bonferroni-corrected t-tests showed that own-gender bias was present with emotional expressions recognized more accurately in female faces (*M* = 71%, *SD* = 22%) than in male faces (*M* = 64%, *SD* = 26%), but only when they look untrustworthy, *t*_(108)_ = 10.77, *p* < 0.001 (see Fig. 2). Age was not found to be a significant covariate (*F* = 0.22, *p* = 0.64, η^2^_p_ = 0.002).

### Reaction times

**Fig. 3:**
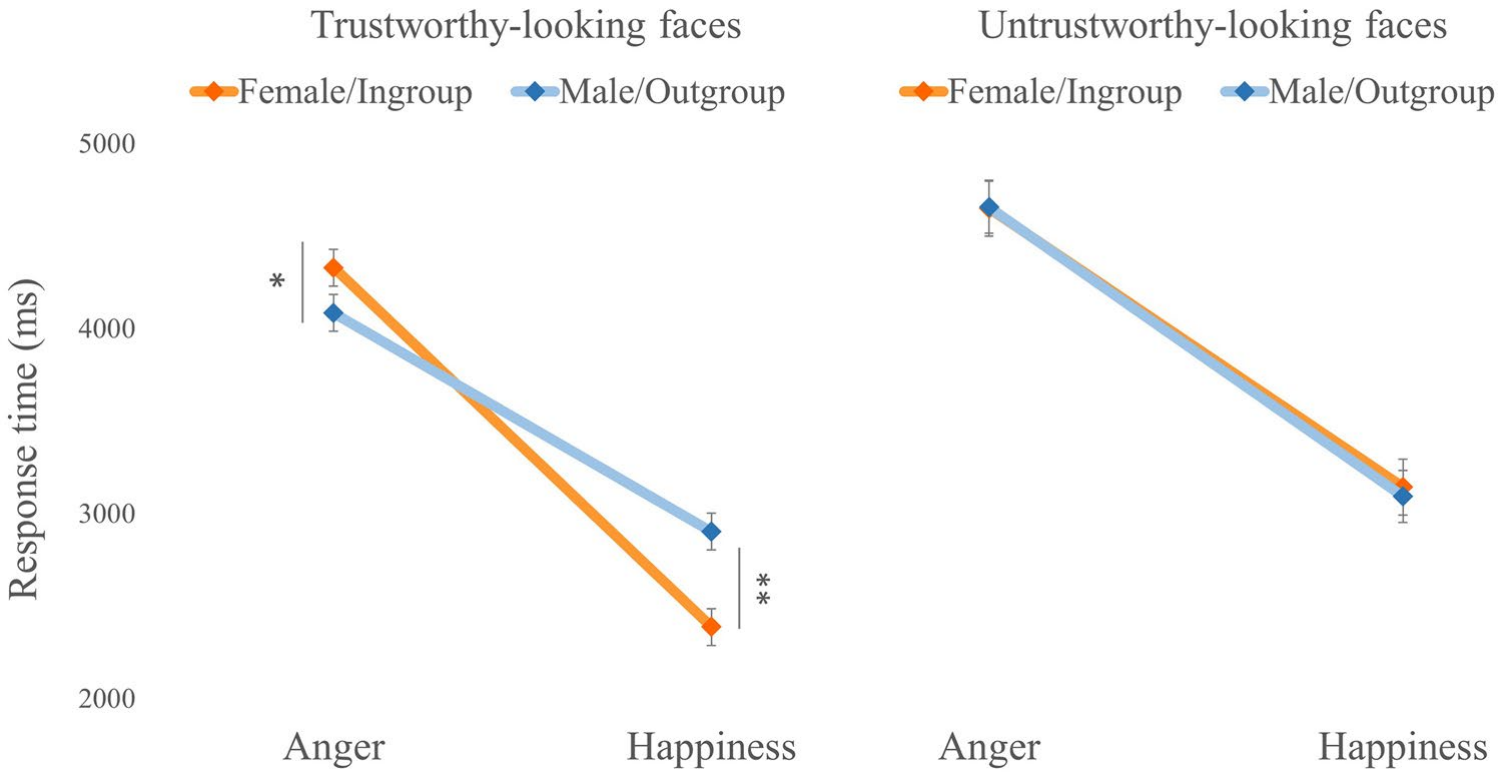
Response time (ms) for the recognition of happiness and anger on female and male targets in trustworthy-looking and trustworthy-looking faces. Error bars represent standard error of the mean. Note. ∗p < .01, ∗∗p < 0.001

Results showed a main effect of Facial Trustworthiness, with reaction times being faster in the trustworthy-looking condition (*M* = 3426.76 ms, *SD* = 1103.49) than in the untrustworthy-looking condition (*M* = 3874.68 ms, *SD* = 1432.37), *F*(1, 108) = 48.24, *p* < 0.001, *η*^*2*^_*p*_ = 0.31. A main effect of Emotion was also observed, *F*(1, 108) = 197.07, *p* < 0.001, *η*^*2*^_*p*_ = 0.65, indicating that happy expressions (*M* = 2878.82 ms, *SD* = 981.50) were recognized faster than angry expressions (*M* = 4422.62 ms, *SD* = 1654.85). As shown in Fig. 3, the 3-way interaction Face Gender × Facial Trustworthiness × Emotion, *F*(1, 108) = 19.07, *p* < 0.01, *η*^*2*^_*p*_ = 0.15, revealed that, only in the trustworthy-looking condition, the recognition of happiness was faster when displayed by female faces (*M* = 2387.64 ms, *SD* = 882.34) compared to male faces (*M* = 2903.52 ms, *SD* = 1016.05), *t*_(108)_ = 62.85, *p* < 0.001, whereas the recognition of anger was faster when displayed by male (*M* = 4085.45 ms, *SD* = 1557.53) faces compared to female faces (*M* = 4330.44 ms, *SD* = 1593.15), *t*_(108)_ = 6.65, *p* < 0.01. Again, age was not found to be a significant covariate (*F* = 0.20, *p* = 0.66, η^2^_p_ = 0.002).

## Discussion

During social interactions, people tend to adapt their behavior to social categories, such as gender, or their evaluation of others in terms of trustworthy-looking appearance (Bagnis et al., 2019; Todorov et al., 2015). Here, our aim was to investigate the intersection of multiple social category dimensions derived by invariant facial features (i.e., gender and facial trustworthiness) in affecting face emotion recognition.

In keeping with our hypothesis, the present results showed that facial trustworthiness can modulate the own-gender bias on emotion recognition. In particular, the own-gender bias was specific for untrustworthy-looking faces (H1b), where emotional expressions were recognized less accurately when displayed by an outgroup member (i.e., an untrustworthy man) than by an ingroup member (i.e., an untrustworthy woman). On the contrary, facial features that convey trustworthiness seems to be able to reduce the own-gender bias (H1a), attenuating differences in recognizing female and male emotional expressions.

These results may be mutually explained by a differential activation of the motivational systems by untrustworthy-looking and trustworthy-looking faces and by the involvement of a different level of attentional resources to the stimuli (Bradley et al., 2001; Oosterhof & Todorov, 2008; Pessoa, 2009; Schupp et al., 2004). Specifically, it has been shown that untrustworthy-looking faces represent social stimuli perceived as potentially threatening and, thus, activate the defensive motivational system associated with evolutionarily preserved avoidance responses (Colonnello et al., 2019a, 2019b; Colonnello et al., 2019a, 2019b). Consistent with an attentional negativity-bias (i.e., an adaptive evolutionary bias that foster negative stimuli detection to avoid threat and danger), it is also possible that untrustworthy-looking faces have captured higher attentional resources, diverting processing away from the main task with effects on emotion recognition (Eastwood et al., 2003; Öhman et al., 2001; Schupp et al., 2004). Consistently, here, emotion recognition was generally worst for untrustworthy-looking faces compared to trustworthy-looking faces. However, the interaction between gender and facial trustworthiness revealed that recognition accuracy was higher when the emotion was expressed by untrustworthy-looking woman (i.e., ingroup) compared to untrustworthy-looking man (i.e., outgroup). This finding suggests that the ingroup category is less susceptible to the sense of threat led by the untrustworthy facial appearance and, thus, less likely to undergo a worsening in the emotion recognition performance, revealing the own-gender bias. In fact, the categorization among humans between “us”, ingroups, and “them”, outgroups, is an adaptive mechanism that allows to maximize our behavioral responses (Brewer, 1999; Paladino & Castelli, 2008). The ability to recognize quickly and accurately the emotional expressions of others have fundamental consequences on social interactions, and, here, the avoidance behavior usually activated by untrustworthy-looking faces seems to be weaken when emotions were displayed by the ingroup category increasing the emotion recognition, in favor of “our” compared to “their” emotional expressions. Moreover, when the emotion is displayed by an untrustworthy-looking face, the differences in familiar facial features and experience level (i.e., greater familiarity with female faces) may have contributed to the more accurate recognition of own-gender emotional expressions. Consistently, own-gender bias in females is thought to be an advantage based on an early perceptual expertise for female faces that is reinforced during social development through reciprocal interactions (Herlitz & Lovén, 2013).

On the contrary, no evidence for a response bias toward ingroup and outgroup emotions expressed by trustworthy-looking faces was found suggesting that positive inferences on trustworthiness may reduce intergroup differences in emotion recognition, probably due to the activation of the appetitive motivational system and, thus, of approach behavior (Todorov et al., 2008; Lang & Bradley, 2010).

Finally, when we took into account the discrete emotional expressions, namely happiness and anger, we found that facial appearance is able to influence the readiness to recognize female and male facial expressions, in function of discrete emotional expressions. Contrary to our hypothesis of a happy-female (H2a) and anger-male (H2b) advantage facilitated by perceptual overlaps between the facial configuration of trustworthy-looking and feminine faces, and untrustworthy-looking and masculine faces, we did not find a recognition advantage for happiness on trustworthy- looking female faces and anger on untrustworthy-looking male faces. Specifically, in line with the visual structural and stereotype-based accounts (Craig & Lee, 2020), the intersection between female and happiness and between male and anger was found to influence emotion recognition speed, but only when faces looked trustworthy, as happiness was recognized more quickly in females and anger in males. In untrustworthy-looking faces, no differences in recognition speed between female and male emotional expressions were found. A possible explanation for this might be that, when positive inferences on trustworthiness are activated, the appetitive motivational system promotes an adaptive evolutionary mechanism about the readiness to perceive anger in males to avoid potential physical threat and happiness in female to gain social support (Becker et al., 2007; Craig & Lee, 2020; Tay, 2015). Untrustworthiness, on the other hand, seems to interfere with this facilitation, again potentially reflecting a higher capture of attentional resources by negative stimuli (i.e., untrustworthy-looking faces), with a resulting more elaborated cognitive processing than positive ones which may have delayed the emotion categorization (Todorov et al., 2008; Lang & Bradley, 2010; Colonnello et al., 2019a, 2019b).

Some limitations of this work must be addressed. First, our sample was limited to female participants, reducing the generalizability of the results and the interpretation of the mechanisms underlying them. Although additional research is needed to address the same pattern of results with male participants, this work represents a first step to understand whether the effect of gender on emotion recognition is moderated by facial trustworthiness. In addition, it is worth to note that it has already been shown that the own-gender bias is more robust and reliable in women (Herlitz & Lovén, 2013; Lovén et al., 2011; Man & Hills, 2016; Rehnman & Herlitz, 2007). Another limitation lies in the lack of implicit or explicit measures of biases and stereotypes (Amodio & Devine, 2006). Accordingly, future studies need to be carried out using measures to characterize personality individual differences in perceiving intergroup members to disambiguate the underlying mechanisms explained by the visual structural and stereotype-based accounts (Craig & Lee, 2020). Finally, our sample consisted of Caucasian participants. Since it has been shown that people have considerable difficulty perceiving emotional expressions from people of different ethnic backgrounds, resulting in lower recognition accuracy (e.g., Chiao & Ambady, 2007), future studies should investigate the effects of ethnic and cultural differences on the recognition of emotions in trustworthy- and untrustworthy-looking faces.

In conclusion, the present work adds to intergroup literatures by examining the extent to which emotion recognition is influenced by the intersection between social categories led by invariant facial features, such as gender and facial trustworthiness. Results suggested that people are indeed influenced by making automatic inferences about trustworthiness during intergroup interactions. This finding is not only theoretically important, but also holds real-world implications, as trustworthiness inferences from faces as well as gender biases have been demonstrated to have an impact on several social contexts (e.g., Bagnis et al., 2021; Bagnis, et al., 2020; Mattarozzi et al., 2017, 2020; Pireddu et al., 2022; Todorov, 2005; Wilson & Rule, 2015). We showed that negative inferences from untrustworthy-looking faces reveal the own-gender bias and thus may contribute, for example, to systemic gender-based disparities in healthcare (Fitzgerald et al., 2019). Similarly, further work should carry out to extend this line of research to other intergroup bias, such as racial bias or age bias. As such, future studies should take into account potential interaction effects between inferences from facial appearance and intergroup biases when investigating their influence both in experimental and ecological settings.

## Data Availability

Data and materials used to perform the experiment are available upon request.

## References

[CR1] Abrosoft Fantamorph Deluxe 5.0. (2011). Retrieved from http://www.fantamorph.com/index.html

[CR2] Amodio DM, Devine PG (2006). Stereotyping and evaluation in implicit race bias: Evidence for independent constructs and unique effects on behavior. Journal of Personality and Social Psychology.

[CR3] Bagnis A, Celeghin A, Mosso CO, Tamietto M (2019). Toward an integrative science of social vision in intergroup bias. Neuroscience and Biobehavioral Reviews.

[CR4] Bagnis A, Caffo E, Cipolli C, De Palma A, Farina G, Mattarozzi K (2020). Judging health care priority in emergency situations: Patient facial appearance matters. Social Science and Medicine.

[CR5] Bagnis A, Cremonini V, Pasi E, Pasquinelli G, Rubbi I, Russo PM, Mattarozzi K (2021). Facing up to bias in healthcare: The influence of familiarity appearance on hiring decisions. Applied Cognitive Psychology.

[CR6] Becker DV, Kenrick DT, Neuberg SL, Blackwell KC, Smith DM (2007). The confounded nature of angry men and happy women. Journal of Personality and Social Psychology.

[CR7] Bradley MM, Codispoti M, Cuthbert BN, Lang PJ (2001). Emotion and Motivation I: Defensive and Appetitive Reactions in Picture Processing. Emotion.

[CR8] Brewer MB (1999). The Psychology of Prejudice: Ingroup Love or Outgroup Hate?. Journal of Social Issues.

[CR9] Chiao JY, Ambady N, Kitayama S, Cohen D (2007). Cultural neuroscience: Parsing universality and diversity across levels of analysis. Handbook of cultural psychology.

[CR10] Colonnello V, Mattarozzi K, Russo PM (2019). Emotion recognition in medical students: Effects of facial appearance and care schema activation. Medical Education.

[CR11] Colonnello V, Russo PM, Mattarozzi K (2019). First Impression Misleads Emotion Recognition. Frontiers in Psychology.

[CR12] Craig BM, Lee AJ (2020). Stereotypes and Structure in the Interaction between Facial Emotional Expression and Sex Characteristics. Adaptive Human Behavior and Physiology.

[CR13] Craig BM, Lipp OV (2017). The Influence of Multiple Social Categories on Emotion Perception..

[CR200] Eastwood, J. D., Smilek, D., & Merikle, P. M. (2003). Negative facial expression captures attention and disrupts performance. *Perception & psychophysics,**65*(3), 352–358. 10.3758/bf03194566 12785065

[CR14] Faul F, Erdfelder E, Lang A-G, Buchner A (2007). G*Power 3: A flexible statistical power analysis program for the social, behavioral, and biomedical. Behavior Research Methods.

[CR15] Fitzgerald C, Martin A, Berner D, Hurst S (2019). Interventions designed to reduce implicit prejudices and implicit stereotypes in real world contexts: A systematic review. BMC Psychology.

[CR16] Freeman J, Johnson K (2016). More Than Meets the Eye: Split-Second Social Perception. Trends in Cognitive Sciences.

[CR17] Harris DA, Ciaramitaro VM (2016). Interdependent mechanisms for processing gender and emotion: The special status of angry male faces. Frontiers in Psychology.

[CR18] Haxby JVJ, Hoffman EEA, Gobbini MI (2000). The distributed human neural system for face perception. Trends in Cognitive Sciences.

[CR19] Herlitz A, Lovén J (2013). Sex differences and the own-gender bias in face recognition: A meta-analytic review. Visual Cognition.

[CR20] Hess U, Adams RBJ, Kleck RE (2004). Facial Appearance, Gender, and Emotion Expression. Emotion.

[CR21] Hess U, Adams RB, Kleck RE (2009). The face is not an empty canvas: How facial expressions interact with facial appearance. Philosophical Transactions of the Royal Society b: Biological Sciences.

[CR22] Hewstone M, Rubin M, Willis H (2002). Intergroup bias. Annual Review of Psychology.

[CR23] Hugenberg K, Sczesny S (2006). Moderates Happy Face Advantage on Wonderful Women and Seeing Smiles : Social Categorization Moderates the Happy Face Response Latency Advantage. Social Cognition.

[CR24] IBM Corp (2020). IBM SPSS Statistics for Windows (Version 25.0.) Armonk, NY: IBM Corp.

[CR25] Jack RE, Schyns PG (2015). The Human Face as a Dynamic Tool for Social Communication. Current Biology.

[CR26] Kret M, Pichon S, Grezes J, de Gelder B (2011). Men fear other men most: Gender specific brain activations in perceiving threat from dynamic faces and bodies-an fMRI study. Frontiers in Psychology.

[CR27] Lang PJ, Bradley MM (2010). Emotion and the motivational brain. Biological Psychology.

[CR28] Lovén J, Herlitz A, Rehnman J (2011). Women’s own-gender bias in face recognition memory: The role of attention at encoding. Experimental Psychology.

[CR29] Lundqvist, D., Flykt, A., & Ohman, A. (1998). Karolinska directed emotional faces [database of standardized facial images]. *Psychology Section, Department of Clinical Neuroscience, Karolinska Hospital, S-171*, *76*

[CR30] Macrae CN, Bodenhausen GV (2000). Social cognition: Thinking categorically about others. Annual Review of Psychology.

[CR31] Man TW, Hills PJ (2016). Eye-tracking the own-gender bias in face recognition: Other-gender faces are viewed differently to own-gender faces. Visual Cognition.

[CR32] Mason MF, Cloutier J, Macrae CN (2006). On Construing Others: Category and Stereotype Activation from Facial Cues. Social Cognition.

[CR33] Mattarozzi K, Todorov A, Codispoti M (2015). Memory for faces: The effect of facial appearance and the context in which the face is encountered. Psychological Research Psychologische Forschung.

[CR34] Mattarozzi K, Colonnello V, De Gioia F, Todorov A (2017). I care, even after the first impression: Facial appearance-based evaluations in healthcare context. Social Science and Medicine.

[CR35] Mattarozzi K, Caponera E, Russo PM, Colonnello V, Bassetti M (2020). Pain and satisfaction : healthcare providers ’ facial appearance matters. Psychological Research.

[CR36] Mill A, Allik J, Realo A, Valk R (2009). Age-related differences in emotion recognition ability: A cross-sectional study. Emotion.

[CR37] Montepare JM, Dobish H (2003). The contribution of emotion perceptions and their overgeneralizations to trait impressions. Journal of Nonverbal Behavior.

[CR300] Öhman, A., Flykt, A., & Esteves, F. (2001). Emotion drives attention: detecting the snake in the grass. *Journal of experimental psychology: general,**130*(3), 466. 10.1037/0096-3445.130.3.466 11561921

[CR38] Oosterhof NN, Todorov A (2008). The functional basis of face evaluation. Proceedings of the National Academy of Sciences of the United States of America.

[CR39] Oosterhof NN, Todorov A (2009). Shared Perceptual Basis of Emotional Expressions and Trustworthiness Impressions From Faces. Emotion.

[CR40] Paladino MP, Castelli L (2008). On the immediate consequences of intergroup categorization: Activation of approach and avoidance motor behavior toward ingroup and outgroup members. Personality and Social Psychology Bulletin.

[CR41] Pessoa L (2009). How do emotion and motivation direct executive control?. Trends in Cognitive Sciences.

[CR500] Pireddu, S., Bongiorno, R., Ryan, M. K., Rubini, M., & Menegatti, M. (2022). The deficit bias: Candidate gender differences in the relative importance of facial stereotypic qualities to leadership hiring. *British Journal of Social Psychology,**61*(2), 644–671. 10.1111/bjso.12501 PMC9293180 34553397

[CR42] Psychology Software Tools, Inc. [E-Prime 3.0]. (2016). Retrieved from https://support.pstnet.com/.

[CR100] Quinn, K. A., & Macrae, C. N. (2011). The face and person perception: Insights from social cognition. *British Journal of Psychology,**102*(4), 849–867. 10.1111/j.2044-8295.2011.02030.x 21988388

[CR43] Rehnman J, Herlitz A (2007). Women remember more faces than men do. Acta Psychologica.

[CR44] Said CP, Sebe N, Todorov A (2009). Structural Resemblance to Emotional Expressions Predicts Evaluation of Emotionally Neutral Faces. Emotion.

[CR45] Schupp HT, Junghöfer M, Öhman A, Weike AI, Stockburger J, Hamm AO (2004). The facilitated processing of threatening faces: An ERP analysis. Emotion.

[CR46] Tay PKC (2015). The adaptive value associated with expressing and perceiving angry-male and happy-female faces. Frontiers in Psychology.

[CR47] Todorov A (2005). Inferences of Competence from Faces Predict Election Outcomes. Science.

[CR400] Todorov, A., Said, C. P., Engell, A. D., & Oosterhof, N. N. (2008). Understanding evaluation of faces on social dimensions. *Trends in cognitive sciences,**12*(12), 455–460. 10.1016/j.tics.2008.10.001 18951830

[CR48] Todorov A, Olivola CY, Dotsch R, Mende-Siedlecki P (2015). Social Attributions from Faces: Determinants, Consequences, Accuracy, and Functional Significance. Annual Review of Psychology.

[CR49] Wacker R, Bölte S, Dziobek I (2017). Women know better what other women think and feel: Gender effects on mindreading across the adult life span. Frontiers in Psychology.

[CR50] Wilson JP, Rule NO (2015). Facial Trustworthiness Predicts Extreme Criminal-Sentencing Outcomes. Psychological Science.

